# Fertility preservation and breast cancer: a review

**DOI:** 10.3332/ecancer.2015.503

**Published:** 2015-02-03

**Authors:** María de Pedro, Borja Otero, Belén Martín

**Affiliations:** 1 Department of Obstetrics and Gynecology, HM Nuevo Belén University Hospital, HM Hospitales, José Silva 7, Madrid 28043, Spain; 2 Department of Obstetrics and Gynecology, Unit of Gynecologic Oncology, Cruces University Hospital, Barakaldo 48903, Spain; 3 Department of Obstetrics and Gynecology, Getafe University Hospital, Getafe 28905, Spain

**Keywords:** breast, cancer, fertility, sparing, preservation

## Abstract

Breast cancer is the most common malignancy in women, and its incidence increases with age, with the majority of patients diagnosed after menopause. However, in 15–25% of cases, patients are premenopausal at the time of diagnosis, and about 7% of them are below the age of 40. Therefore, a considerable amount of young women are diagnosed with breast cancer during their reproductive life. Within this group, most cancer cases require cytotoxic chemotherapy and/or hormone therapy, which are responsible for a decrease in the patients’ reproductive function, along with their age. The efficacy of such treatments, among other factors, has led to a high five-year-survival rate, which results in an increasing number of young women who survive breast cancer before having fulfilled their reproductive wishes, especially considering the current trend to delay pregnancy until the late 30s or early 40s in developed countries. The combination of these factors justifies the importance of fertility preservation and reproductive counselling at the time of breast cancer diagnosis in young women.

A wide range of fertility preservation techniques has been developed, such as ovarian suppression, oocyte and embryo cryopreservation, immature oocyte retrieval and *in vitro* maturation, and ovarian tissue cryopreservation.

Early counselling and referral of these patients to fertility specialists are fundamental factors in order to maximise their chances of pregnancy. This review aims to update the knowledge about the influence of breast cancer in fertility, the influence of pregnancy and fertility preservation techniques in breast cancer patients and assessment of ovarian reserve for a better treatment choice. A special section dedicated to BRCA-mutation carriers has been included because of their specific features.

A comprehensive literature search has been conducted, including publications from the last five years.

## Fertility preservation and breast cancer: a review

### Introduction

Breast cancer is the most common malignancy in women and a considerable amount of cases are detected during their reproductive life. The implications of breast cancer in young women (disease aggressiveness with need for systemic therapy in most cases, high five-year survival rate) and current trends in family planning in developed countries (deliberate delay of pregnancy until the late 30s or early 40s) make fertility preservation a necessary issue to attend to at the time of diagnosis. Recent improvements and growing research in fertility preservation techniques have made a number of possibilities available for these women. Unfortunately, both patients and health care providers often forget to attend to this issue after diagnosis, where an early referral to a fertility specialist is fundamental in order to achieve the best results.

### Material and methods

This review aims to update the knowledge about the various aspects of breast cancer and fertility preservation. In order to do so, a PubMed database search was conducted, using the terms ‘BREAST, CANCER, FERTILITY, SPARING, PRESERVATION’. To ensure that only the latest evidence was included, papers published before 2011 were not considered. Since a considerable amount of those publications are literature reviews, many of the references corresponded to older articles.

### Conclusions

Early diagnostic methods, targeted therapies, and prolonged survival rates have made fertility preservation a major issue when treating young breast cancer patients. However, this department is often considered secondary, when early referral and treatment design are crucial to its success. Not only could most young women diagnosed with breast cancer benefit from a wide variety of fertility preservation techniques such as ovarian suppression, oocyte and embryo cryopreservation, immature oocyte retrieval and *in vitro* maturation, and ovarian tissue cryopreservation, but they could resort to these treatments without compromising the efficacy of their anticancer therapy. Individual characteristics should be taken into account, especially the molecular subtype of breast tumour, which determines the need for gonadotoxic therapy, and the ovarian reserve at the time of diagnosis. Finally, BRCA mutation carriers, either healthy or already diagnosed with breast cancer, are also capable of achieving pregnancy despite their added difficulties—mainly a higher tumour aggressiveness and a lower ovarian reserve.

## Breast cancer incidence in premenopausal women

### Data on breast cancer incidence according to age

Breast cancer is the most common malignancy in women and its incidence increases with age, with the majority of patients diagnosed after menopause [1]. However, in 15–25% of cases, patients are premenopausal at the time of diagnosis [2], and about 7% of them are below the age of 40 (www.seer.cancer.gov.2008) [3].

### Breast cancer survival in young patients

Despite being considered as a different entity, with a poorer prognosis than postmenopausal breast cancer [4–8], five-year survival for breast cancer patients younger than 40 years of age in the United States has increased from 75.2% in the 1970s to the current 86.9% [9].

### Systemic breast cancer therapy

Part of that high survival rate is because of the efficacy of current breast cancer treatment. A cytotoxic chemotherapy regimen is virtually mandatory for all patients presenting with histological grade 3 tumours, high Ki-67, low hormone receptor status, HER-2 positivity or triple-negative status, high 21-gene recurrence score (RS), high-risk 70-gene signature, and the involvement of more than three lymph nodes [10]. Since about two-thirds diagnosed below the age of 40 present with stage II breast cancer or higher [11], it can be safely stated that a considerable number of these patients will undergo cytotoxic chemotherapy, which is at the least partly responsible for the low live birth rate after diagnosis. Moreover, it has been demonstrated that young patients benefit further from chemotherapy compared to older patients [12].

In addition to chemotherapy, about two-thirds of patients under 40 generally present with a hormone receptor positive tumour and will therefore receive a ten-year treatment regimen with tamoxifen with or without a GnRH agonist.

### Current trends in family planning

Women in developed countries show a rising trend to delay pregnancy until the late 30s and early 40s (almost 25% of first live births in the United States occur between the ages of 30 and 40) which results in many breast cancer patients who have not fulfilled–or even thought about– their reproductive wishes at the time of diagnosis [13].

There is some controversy about the amount of breast cancer patients who pursue a pregnancy after diagnosis and about the reasons for their choices. Survey data [14] suggest that approximately half of women who survive breast cancer wish or try to become pregnant, but the fact is only about 10% of women who develop invasive breast cancer before 40 years of age have children after diagnosis [15–17], even though observational studies in women who became pregnant after diagnosis have not shown a higher risk of relapse or death [18–20]. Personal fears and also lack of appropriate and rapid fertility counselling probably contribute to the low pregnancy rate in these patients. In fact, an American survey reported that only around 50% of cancer survivors recalled having being counselled by their doctors on how cancer treatment would impact their fertility [21, 22].

### Summary

− There is a considerable number of young women who are diagnosed with breast cancer during their reproductive life.
− Within this group, most cancer cases require cytotoxic chemotherapy and/or hormone therapy, which are responsible for a decrease in these patients’ reproductive function, along with their age.
− The efficacy of those treatments, among other factors, has led to a high five-year-survival rate, which results in an increasing number of young women who survive breast cancer before having fulfilled their reproductive wishes, especially considering the current trend to delay pregnancy until the late 30s or early 40s in developed countries.
− The combination of these factors justifies the importance of fertility preservation and reproductive counselling at the time of breast cancer diagnosis in young women.

## Fertility after breast cancer treatment

Fertility can be impaired in breast cancer patients for various reasons, mainly age at diagnosis, use of gonadotoxic chemotherapy, and duration of endocrine treatment, with the subsequent need to delay pregnancy.

### Age at Diagnosis

Healthy women undergo a progressive decrease in oocyte population from fetal life to menopause [23], and they are considered to be sterile about five to ten years before their last menstrual period [24]. By the age of 37 years, more than 95% of the oocytes present at birth have already undergone apoptosis. By the age of 40 years, fecundity rate is 50% compared to that of a 30-year-old woman, with a three-fold higher risk of miscarriage [25].

In order to better assess individual ovarian reserve, two basic examinations are available: the concentration of anti-Mullerian hormone (AMH) and the ultrasonographic antral follicle count [26].

Given that most premenopausal breast cancer patients are older than 35, it is easy to understand how this group present with a natural baseline fertility impairment because of their age and how cancer condition and anticancer therapies accelerate the decrease in their reproductive function.

### Cancer-related factors

Cancer itself lowers AMH and inhibine-B levels and elevates follicle stimulating hormone (FSH) concentration compared to healthy women [27], especially in women who present chemo-induced amenorrhoea (CIA)–in which case, these change seem to precede CIA [27, 28], while those patients who kept their cycles during treatment show milder modifications. There is no agreement yet as to the importance of hormone levels or antral follicle count prior to treatment in terms of regaining menstrual cycles [29, 30].

### Treatment-related factors

However, the critical effect of breast cancer on fertility derives from adjuvant or neoadjuvant treatments, mainly chemotherapy but also hormonal blockade. Although its effects may alter ovarian reserve and endocrine function in different ways and to a different extent in the same patient, some of the changes they induce are rather constant and will be discussed below. As a general rule, toxicity of different protocols shows effects only during the recovery phase, not at onset of treatment [1].

Choice of adjuvant treatment is made according to the risk of recurrence and the type of tumour. While the 15-year risk of recurrence and death for women under 50 not receiving adjuvant systemic therapy is 53% and 42%, respectively, administration of adjuvant therapy reduces those rates by half, considering all stages of disease and all molecular types of tumour [31]. When analysed individually, these figures differ significantly, with a 12.5% breast cancer mortality rate at 15 years for women under 50 with low-risk node-negative tumours, 25% for women with high-risk node-negative tumours, and 50% for node-positive tumours [31].

As previously stated, according to stage, adjuvant chemotherapy is required for many patients presenting with stage II tumours and it is also recommended for stage I patients with a tumour larger than 1 cm [10]. These two groups together account for more than two-third of patients [11]. On the other hand, neoadjuvant chemotherapy is usually indicated for patients at stage III.

With regard to molecular characteristics, 15% of all breast tumours are luminal A, 60% are luminal B, and the rarer types such as HER-2+ and basal-like tumours each account for 10–15% of cases [32, 33]. The most common types of tumours in young women are also luminal B and luminal A. However, HER-2+ and basal-like tumours are more frequent in this age group than in older women. Standard adjuvant regimen for young women with luminal A tumours includes hormonal blockade during ten years—typically with tamoxifen—with or without ovarian suppression, either medical with GnRH agonists or surgically by bilateral adnexectomy. The additional benefit of chemotherapy in these patients remains unclear, although it is widely used in the United States, especially for women younger than 40. On the other hand, most patients diagnosed with luminal B tumours receive chemotherapy [32–33]. In case of hormone receptor negative tumours larger than 1 cm, or smaller than 1 cm but presenting high histological grade and/or HER-2 positivity, chemotherapy is equally offered. Finally, HER-2+ patients receive HER-2-directed chemotherapy for 12 months [34].

Upon diagnosis, referral for fertility preservation occurs between primary surgery and start of adjuvant chemotherapy. In case of neoadjuvant therapy, such referral should take place before treatment starts. Not only does this provide a very limited time frame but also presents the risk of tumour growth during follicle stimulation, a particularly delicate matter for patients with ER+ tumours.

### Chemotherapy-Induced gonadotoxicity

Chemotherapeutics induces ovarian toxicity mainly by damaging primordial oocytes, granulosa cells, and ovarian stroma [35]. They have also been found to cause significant vascular injury, including thickening and proliferation of cortical vessels, focal cortical fibrosis, and segmental collagen deposition [36].

All chemotherapeutics, irrespective of their action mechanism, can damage developing follicles by interrupting granulosa cell development, which subsequently causes amenorrhoea. Since new follicles develop from intact primordial follicles within a lapse of three to six-months, this amenorrhoea tends to be transient for non-DNA-damaging agents, with no effects on follicle reserve. On other hand, amenorrhoea has been linked to a reduction of recurrence and to an improved survival [37–40], as it can be the reflection of a permanent ovarian dysfunction. Given the hormonal component of most breast tumours, this gonadotoxicity would interfere with breast carcinogenesis.

The most important predictive factors for chemo-induced amenorrhoea are age, dose, and type of chemotherapy, particularly the number of cycles of alkylating agents and exposure to anthracyclines, taxanes, and platinum analogs [41–44].

#### Age

Women older than 40 are at five-fold higher risk of chemo-induced amenorrhoea (CIA) than younger women [45]. However, women younger than 30 still present a CIA rate ranging from 15 to 40% [46]. These figures vary dramatically across studies, mainly because of high heterogeneity in how amenorrhoea is defined—some papers consider time lapses from 3 to 12 months—but also because of different study populations and different protocols. For instance, studies including very young population tend to yield CIA rates as low as 17% [47]. However, a particular paper regarding a group of premenopausal patients where only 25% were younger than 40, obtained a CIA rate higher than 90% [48]. On the other hand, 15–50% of patients diagnosed below age 40 will recover menses, as opposed to only 10% of patients over 40 and 5% over 45 [45, 47, 50, 51].

It must also be noted that despite its wide use as markers of reproductive function, neither amenorrhoea is a synonym of infertility, nor resume of menstrual cycles is a sign of fertility [50].

#### Impact of chemotherapeutic agents

The most significant agent in terms of loss of ovarian function is cyclophosphamide. Cyclophosphamide is an alkylating agent with demonstrated efficacy as adjuvant treatment for breast cancer and it has long been used with this purpose. However, it has a strong impact in primordial follicle reserve: a reduction higher than 90% in follicle density has been reported 48 hours after administration [51]. The effect on reproductive life span is equally strong: one single cycle at a standard dose can accelerate ovarian ageing by up to three years in terms of reproductive function, while a whole regimen administered over 12–16 weeks can increase that ageing by up to ten years [41–43]. This means that a woman who starts at 30 will have the equivalent ovarian age of 40 by the end of the treatment.

Most current regimens add a taxane to anthracycline and cyclophosphamide for all but luminal A tumours [33, 52]. Overall, a 3% absolute increase in survival is observed if taxanes are added to an anthracycline and cyclophosphamide regimen [53]. The impact of taxanes on the primordial follicle population still remains unclear.

**Figure 1.**
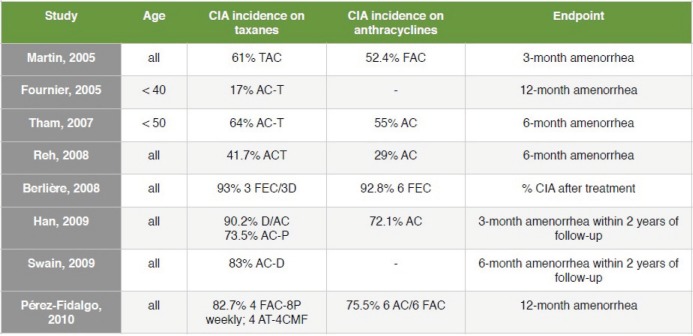
CIA incidence reported by different studies.

Adapted from Mailliez A, Decanter C and Bonneterre J (2011) **Adjuvant chemotherapy for breast cancer and fertility: estimation of the impact, options of preservation and role of the oncologist** *Bull Cancer* **98**(7) 741–51

With regard to the effect of specific chemotherapy protocols on ovarian function, a high data heterogeneity is observed. The range of CIA rates for anthracycline-based protocols is as wide as 29–93%, and 17–93% for taxane plus anthracycline regimens [2]. A recent prospective study observed similar CIA rates for different protocols such as cyclophosphamide, methotrexate, fluorouracil (CMF), AC (doxorubicin and cyclophosphamide), ACT (doxorubicin, cyclophosphamide and paclitaxel), and AC plus docetaxel, although patients treated with CMF and AC plus docetaxel were less likely to resume their menses [54]. Figure 1 summarises CIA rates resulting from different studies.

Neither trastuzumab, which improves relapse-free and overall survival by about 50% in women with HER-2+ tumours [34], or other HER-2-directed agents, have yet shown ovarian toxicity [55].

#### Strategies to reduce follicular damage

Chemotherapy regimens can usually be altered somewhat to reduce gonadotoxicity. As an example, for luminal B tumours, three cycles of fluorouracil, epirubicin, cyclophosphamide (FEC) followed by three cycles of docetaxel has proven equally effective as six cycles of FEC with less ovarian damage because of reduced amount of cyclophosphamide [48]. It has also been suggested that taxane and carboplatin are as effective as anthracycline, cyclophosphamide, and taxane combinations for most HER-2 positive tumours [56, 57], and probably less likely to result in sterility, since, as previously explained, cyclophosphamide is the main responsible for gonadotoxicity. However, no information on fertility is provided by such trials. Triple negative breast tumours often present multiple defects in DNA repair mechanisms, and women with these tumours may benefit from treatment with cis- or carboplatin regimens in combination with poly ADP ribose polymerase (PARP)-inhibitors [58], although this promising hypothesis still needs further studies.

### Endocrine treatment

Few data are available about the direct ovarian toxicity of tamoxifen. Lower concentrations of AMH and higher levels of FSH have been observed in women who maintained their menses during treatment [59] but data are inconclusive. The main problem with tamoxifen is its prolonged treatment duration. The standard 20 mg/day for ten years may significantly reduce the reproductive chances of a breast cancer patient diagnosed after 35 years of age, just for the physiological reduction of the oocyte quantity and quality that occurs with ageing. Innovative protocols with shorter treatment duration are ongoing or have been proposed [60].

## Evaluation of the ovarian reserve

The evolution and improvement of anticancer treatments have resulted in a high prevalence of young breast cancer survivors for whom preservation of future fertility is likely to be a priority. Primordial follicles begin to decrease in number from early stages in the embryonic period until definitive menopause. This fact marks the importance of estimating the ovarian reserve of patients that undergo anticancer therapy.

First of all, the question rises whether breast cancer can somehow affect the ovarian reserve. Several studies have demonstrated that this tumour has no influence on the ovarian reserve as patients developing post-treatment amenorrhoea have pretreatment anti-müllerian hormone (AMH) levels lower than age adjusted controls [27]. There are several methods that have been investigated as valid ovarian reserve estimators, including clinical characteristics, ultrasonographic tests, and hormonal markers. If we just consider clinical features of these patients, we should take into account that the presence of regular menses in a patient after receiving chemotherapy does not mean she is fertile and vice versa [61]. Regarding ultrasonographic tests, both measurement of ovarian volume and quantification of antral follicles had been used without great accuracy as ovarian reserve predictors. Finally, hormonal markers can be useful to asses a patient’s ovarian reserve. Different relations between ovarian reserve and FSH, estradiol (E2), inhibine B, and AMH have been investigated. Determination of these hormonal markers may mean two problems. Firstly, some of them have cyclic variations which can lead to the need of serial determinations in order to obtain an estimate of the ovarian reserve. On the other hand, hormonal levels can vary in premenopausal breast cancer patients in tamoxifen therapy [62]. AMH has shown to be the most reliable marker both in general population and in breast cancer patients.

### Antimüllerian hormone

AMH is produced in the antral follicles in the ovary and so AMH concentration can parallel the number of these follicles and therefore reflect the ovarian reserve of women. Serum levels of AMH remain much more stable during the menstrual cycle than other parameters such as FSH, Inhibine E or E2 which tends to continuously decrease during women’s life [63]. This last characteristic means that even in prepuberal girls, AMH can be a good predictor of ovarian reserve when gonodotoxic treatments are needed, as is the case in patients with leukaemia [64].

These facts have also led to the determination of cut-off values in general population that can predict the success of assisted reproduction techniques (ART). These cut-off values have also been validated in breast cancer patients showing that levels>1.2 ng/mL prior to the beginning of chemotherapy is associated with higher possibilities of obtaining more than four mature oocytes that could later be used for different ART [65]. Pre-chemotherapy AMH levels also predict long-term ovarian function [66].

This parameter being the most reliable predictor of ovarian function, it should never be considered as definitive, as pregnancies with very low or undetectable levels of AMH have been reported [67].

## Fertility preserving techniques

Once a good ovarian reserve has been predicted, several ARTs can be used. These techniques could be divided into three groups: techniques that try to reduce the impact of chemotherapy on ovarian function, techniques finalised to obtain oocytes or embryos that could be cryopreserved, and techniques which try to preserve and freeze ovarian tissue before the beginning of the chemotherapy.

### Ovarian suppression

Several studies have shown that the older a woman is when receiving chemotherapy the higher the amenorrhoea rate she will have [46, 68]. This difference exist if we compare pre-puberal and post puberal women, and this difference could mean that a suppressed ovary could have some kind of protection against chemotherapy [69].

In order to achieve this, ovarian suppression and consequent GnRH agonists (GnRHa) have been investigated. Although experimental studies in animals have demonstrated a protective effect against chemotherapy experiments in humans have shown inconsistent results leading the American Society of Clinical Oncology to conclude that: ‘Given the current state of knowledge regarding these agents, it is the opinion of the Update Panel that GnRHa is not an effective method of fertility preservation. Furthermore, complete ovarian suppression is not achieved for several weeks after administration. However, there may be other potential benefits such as inhibiting menses during intensive chemotherapy, thus preventing complications such as menorrhagia’.

### Oocyte and embryo cryopreservation

As, the protective effect of these GnRH agonists could only be certified after the end of chemotherapy, obtainment of oocytes prior to the beginning of chemotherapy could be a safer option for these patients. As most types of breast cancer are known to be hormone sensitive the elevated E2 serum levels usually obtained in controlled ovarian hyper-stimulation (COH) cycles could be a main problem in order to warrant their oncological prognosis. Several COH protocols have been investigated in order to solve this problem.

#### Tamoxifen

Tamoxifen is a well-known selective estrogen receptor modulator with antagonist effect on the breast. It is widely used in breast cancer patients as it has demonstrated a reduction in overall mortality and relapse rates when used in treatment [71]. The fact that this effect has been demonstrated in premenopausal women with E2 serum levels similar to fertile women has led to the investigation of the protective effect of this drug in breast cancer patients undergoing COH. A recent study has demonstrated that there are no statistical differences in the number of oocytes collected between patients when tamoxifen is co-administered during COH compared to those patients which did not receive this drug [72].

#### Aromatase inhibitors

Similarly to tamoxifen, aromatase inhibitors have shown to decrease E2 serum levels in breast cancer postmenopausal patients being effective in reducing breast cancer mortality and relapses [73].

This ability to keep E2 levels in a low range have also led to the investigation of their role in keeping low E2 levels in breast cancer patients undergoing COH. Letrozole, has shown to be safe in these patients even leading to the possibility of having two COH cycles before starting chemotherapy thus getting more oocytes in these patients [74]. On the other hand, it has shown that this treatment could also lead to the achievement of higher rates of immature oocytes [75].

#### GnRHa versus hCG

GnRH agonists have been widely used as an alternative to hCG in order to trigger oocyte ripening during COH. The rationale of this particular technique is that the lower hyper-stimulation syndrome that occurs with this medication could be very useful in breast cancer patients undergoing COH. Several studies have investigated this theory and have demonstrated that when GnRH agonists are used on these patients, fewer ovarian hyper-stimulation syndrome occurs and that higher number of oocytes are retrieved along with higher maturation and fertilisation rates [76].

### Immature oocyte retrieval and in vitro maturation

A novel technique has been developed in recent years consisting in the retrieval of immature oocytes followed by *in vitro* maturation. This technique has several advantages as the possibility of obtaining these oocytes in unstimulated ovaries thus being faster than starting a COH. Furthermore, E2 levels would keep in low ranges on these patients. Finally the cost of this treatment would be lower as very less medication is needed to obtain the oocytes [77]. These techniques can be used as mentioned before in unstimulated ovaries or in addition to COH. Either way it seems that 50% of the immature oocytes retrieved could be maturated in order to be fertilised [78, 79].

### Ovarian tissue cryopreservation

Finally, cryopreservation of ovarian tissue has been investigated. This technique consists on the surgical retrieval of ovarian tissue including whole ovarian cortical tissue. Once this ovarian tissue is obtained it can either be used for post-chemotherapy transplantation or for follicle aspiration in order to get mature or immature oocytes that could be used for one of the previously described techniques.

Once experimental, this technique has nowadays been demonstrated to be useful as reports of the achievement of several pregnancies after ovarian transplantation is being published [80]. This particular technique should be avoided in BRCA mutation carriers in which it could end up with the development of an ovarian cancer.

## Fertility related-knowledge, decision making in women with breast cancer, and role of the health care provider in strategies to preserve fertility

Survival improvement for women with breast cancer and increase of the age to attempt pregnancy because social changes, set the focus on survivorship issues, including fertility. Young survivors of cancer do not only want to preserve their lives, but also their quality of life.

Fertility preservation is an important concern of many young women diagnosed with breast cancer and this fact has been reported in numerous studies [81, 82]. A recent Swedish study reported that only 48% of young female survivors of different cancers were informed about the risks to fertility, only 14% were informed about fertility preservation techniques, and only 2% used these techniques [83].

Misinformation by health care providers is an important issue. An online survey of medical oncologists, surgical oncologists, and clinical nurse specialists in the United Kingdom revealed that many of them are uncertain about fertility preservation strategies [84].

Young women with breast cancer are forced to quickly make important decisions about cancer treatment and fertility preservation. The ideal time to provide information is before initiating treatment with radiation, chemotherapy, or endocrine agents. Unfortunately, many reports have shown that these patients do not receive much information about the effects of these treatments on fertility or options for fertility preservation until the treatment has been started or completed [85, 86].

Peate *et al* reported that women with higher levels of fertility-related knowledge had lower levels of fertility-related decisional conflict. Improvements in patient knowledge may reduce uncertainty about fertility treatment options, which may increase decision quality and informed choice [83]. In the long term, informed choice improves psychological adjustment to breast cancer.

They found that single women and those who are not sure to have children may also wish to discuss fertility preserving strategies before treatment of breast cancer. Currently there are some advocacy groups regarding fertility education and support for survivors. Thanks to their dedicated efforts, information and support for these patients have been widely disseminated through the websites and social media. Fertile Hope, the Young Survival Coalition, Living Beyond Breast Cancer, and the Susan G. Komen for the Cure are advocacy organisations which provide excellent, reliable and evidence-based options and information regarding fertility preservation.

The 2006 American Society of Clinical Oncology (ASCO) Fertility Preservation Guidelines highlighted the need for the following: having frank discussions of fertility preservation, early referral to reproductive specialists, addressing fertility preservation as early as possible before starting cancer treatment, referring for psychosocial specialists if distress is present, and encouraging participation in clinical studies and registries when appropriate. The guideline was updated in 2013 [87]. They reconfirmed the recommendations, adding two significant changes: oocyte cryopreservation was no longer experimental, and replaced the term oncologist with health care provider, assuming that not only oncologists but other physicians, nurses, psychologists, play a vital role in the interdisciplinary approach of fertility preservation.

Receiving a diagnosis of breast cancer may be devastating for young women. This is especially true if they have not completed childbearing plans. It is the responsibility of the health care team to explain prognosis, treatment options, and potential toxicities and adverse effects of chemotherapy, radiotherapy, and endocrine treatments, educate about fertility issues, and refer them early to appropriate specialist as requested. An interdisciplinary approach including those with medical oncologists, reproductive specialists, obstetrician and gynecologists, primary care physicians, nurses, psychologists, and other allied health professionals is ideally used throughout each young breast cancer survivors’ journey. This discussion should be taken as soon as possible to provide wider options for these patients.

## Risk of pregnancy and lactation after breast cancer

### Risk of breast cancer after pregnancy and lactation in the general population

There is evidence that suggests a transient increase in breast cancer in the four years following a pregnancy [88, 89]. However, other authors did not find this relation [90, 91]. Observational data indicate that nulliparous women have a lifetime risk higher than those who have completed one or more pregnancies [92].

Regarding lactation, there are consistent data that reveal a protective effect against breast cancer [93–95] or at least a neutral one [96, 97]. This beneficial effect is more evident if the duration of lactation lasts 24 months during a woman lifetime and if it begins at a young age [94, 95].

### Pregnancy after breast cancer

Recent studies have reported that 40–50% of women with previous history of breast cancer may wish to have a subsequent pregnancy, but only 4–7% manage to conceive [98, 99].

Estrogens are known to play a role in breast carcinogenesis and are increased during pregnancy. For this reason, in the past it has been assumed that a pregnancy after breast cancer may contribute to breast cancer recurrence and a poorer prognosis. These uncertainties have contributed to physicians to advise patients against pregnancy. Some studies reported induced abortion rates about 30% [20, 100].

However, recent available data do not only report an adverse effect of a subsequent pregnancy on breast cancer outcome but also a potential favourable impact on prognosis [60, 101]. Medical literatures have not shown a higher proportion of distant metastases in women who have given birth after breast cancer compared with women with breast cancer who did not become pregnant. Similarly, overall survival in patients treated for breast cancer who subsequently become pregnant compares favourably with controls [102, 103].

A study from the MD Anderson Cancer Centre showed that women in the subgroup of postcancer pregnancy were more likely to have an early stage cancer, negative nodes, and negative hormone receptors, therefore this good prognosis may be the result of a self-selection bias called ‘the healthy mother effect’ [15, 20, 104].

Regarding the subgroup of patients with an endocrine sensitive breast cancer, Azim *et al* found neither a detrimental effect nor DFS (disease free survival) nor OS (overall survival) in women conceiving after breast cancer treatment compared to those who did not. Abortion had no effect on breast cancer outcomes, hence these authors conclude that interruption of pregnancy should not be promoted for therapeutic purposes [105].

Women treated for breast cancer and who wish to become pregnant should be counselled that pregnancy is possible and does not seem to be associated with a worse prognosis for their disease. Pregnancy after breast cancer should not in principle be discouraged. Nonetheless, they have to be aware that the evidence that supports that advice is relatively poor, based on retrospective studies often carrying numerous biases [106]. However, it is important to note that it is not possible to address the impact of subsequent pregnancy on breast cancer prognosis in prospective randomised trials, therefore physicians should have to rely on results from large, well-constructed retrospective studies.

A fully oncologic evaluation should be performed before trying conception, depending on the individual risk of relapse. Patients should be informed about the possibility of breast cancer recurrence even many years after diagnosis.

### Timing of pregnancy for breast cancer survivors

There is no evidence to recommend a time frame from diagnosis to pregnancy. Moreover, recurrence patterns vary according to the molecular subtype of breast cancer: while ER- disease tends to recur within the first two or three years after diagnosis and treatment, luminal-type disease is prone to late relapses, sometimes after five years. Although it would seem reasonable to postpone pregnancy beyond the most likely period of relapse, depending on the molecular subtype, there is no strong evidence that conception prior to that time worsens prognosis. A recent study suggests that women with localised disease, early conception six months after completing treatment is unlikely to reduce survival [20]. In cases of higher risk of early relapse some experts recommend avoiding pregnancy in the two years following diagnosis. If the patient has axillary node involvement, there is low evidence based on expert opinion that recommends waiting five years from diagnosis to attempt a pregnancy [106].

In hormone positive breast cancer patients, women should be fully informed about the risk of stopping tamoxifen prematurely (the earlier the interruption, the higher the risk of relapse). These risks must be balanced with the risk of infertility because of ageing. For the moment, it is recommended to complete endocrine therapy after pregnancy if it happens [107]. Some experts recommend waiting at least four–six months from the end of chemotherapy and the attempt to conceive. The interval recommended after the end of endocrine treatment is at least three months. During primary cancer treatment and this period, it is recommended that safe, non-hormonal contraception should be used. The contraception approaches include barrier methods (male or female condoms, diaphragm, cervical cap), spermicides, sponge, and the copper intrauterine device (IUD).

Due to the lack of strong evidence, caution and individualised and informed decision making are encouraged.

### Risk for the pregnancy and fetus

Data regarding pregnancy and fetal outcome after breast cancer treatment are reassuring. Although chemotherapy has been shown to increase miscarriage frequency, some studies reported that children born from mothers who have received chemotherapy seem not to be in higher risk for congenital defects compared with the general population. Endocrine therapy does not increase the risk of congenital defects after completion of the treatment. In contrast, Dalberg *et al* reported an increased age adjusted risk of preterm birth (relative risk (RR) for gestational age <32 weeks: 3.2, 95% confidence interval (CI) (1.70, 6.03)) and malformations (RR 168, 95% CI (1.11-2.54)), especially in the period of births between 1988–2000, suggesting a role of chemotherapy and hormonal therapies that have been increasingly used over the time. The malformations observed were cardiac defects, urogenital defects, ear malformations, congenital hydrocephaly, and orofacial cleft [108]. Data from other authors indicate that infants born to survivors of breast cancer do not have an increased risk of low birth weight or birth defects when compared to the general population [60, 109].

Young breast cancer survivors should be afraid about the risk of the infant to develop cancer. Data do not suggest that the children of women treated for breast cancer carry an increased risk of cancer except when a genetic cancer syndrome such as BRCA mutation has been identified [110]. Therefore, an appointment with a geneticist should be considered prior to cryopreservation to identify potential genetic risks to the fetus. Preimplantation genetic diagnosis (PGD) can identify embryos without the BRCA defect, thus allowing to select BRCA negative embryos.

There are no specific antenatal guidelines available for survivors of breast cancer who become pregnant. They should be followed up in a multidisciplinary approach.

Before pregnancy is initiated, a complete check-up should be done including clinical breast examination, mammography, ultrasound scan, and, if necessary, magnetic resonance imaging (MRI). If the patient received anthracyclines, a subclinical cardiomyopathy may exist [111]. Pregnancy increases myocardial function and may be complicated by heart failure in the case of a pre-existing dysfunction, although reassuring data have been reported in survivors giving birth after treatment with anthracyclines in their childhood [112]. Therefore, a cardiac ultrasound should be systematically performed prior to an attempt of a pregnancy in this subgroup of patients in order to evaluate the left ventricle ejection fraction. This cardiac risk is increased in patients who have received radiotherapy of the left breast.

During pregnancy, the patient should be examined regularly. Careful breast examination and further explorations should be carried out in case of suspicion of relapse, because estrogens during pregnancy could accelerate tumour growth.

## Breastfeeding after breast cancer

There is no epidemiological data on the impact of breastfeeding on the risk of a second breast cancer or the risk of recurrence in the ipsilateral breast [106]. But there is no evidence that breastfeeding increases the risk of breast cancer recurrence or a second breast cancer developing.

Breastfeeding after breast cancer is not contraindicated for women who do not show any evidence of residual tumour and should be supported with adequate information and counselling [113, 114].

There is no evidence that milk from a mother previously treated for breast cancer increases the risk of disease in the child.

Breast cancer treatment may impair the capability of nursing because of the reduction of milk production related to surgery and radiotherapy.

The periareolar incision often used for cosmetic purposes may reduce the quantity of milk if several ducts have been damaged. If the lesion is central, successful lactation is less likely to be possible. Radiation therapy may negatively influence the function of the treated breast, it induces perilobar and periductal fibrosis, and stenosis of the lactiferous ducts. In addition, the elasticity of the nipple may be impaired, creating difficulty for the suction. If a unilateral total mastectomy has been performed, or in the case of compromised milk production from the treated breast, the woman should be advised that breastfeeding is possible and safe for the infant from a single breast [107].

## Special considerations for BRCA-mutation carriers

A special section must be dedicated to women with BRCA mutations, which present specific issues that must be attended to.

Approximately 10% of breast cancer cases are because of germ-line mutations in susceptibility genes, especially BRCA 1 and BRCA 2. It is estimated that, in the general population, one in every 1000 women is a carrier of BRCA mutations with an increased incidence in certain ethnic groups. BRCA 1/2 mutation carriers have an even higher risk for developing a second primary breast cancer [115–117]. Both prophylactic bilateral salpingo-oophorectomy BSO and tamoxifen use have been shown to decrease the risk of a second primary [118]. BRCA 1 mutation carriers are at a 50–80% lifetime risk of breast cancer, 40–50% risk of developing a second primary breast cancer [115–117] and 40–60% risk of ovarian cancer. Women with BRCA 2 mutations also present a high risk of breast cancer, although the risk of ovarian cancer is lower (10–20%) [118–120]. Therefore, prophylactic bilateral mastectomy and especially BSO are offered to BRCA mutation carriers, since BSO has demonstrated a reduction in ovarian risk close to 95% [121, 122], a decrease in risk of a second primary breast cancer [117], and in short term mortality [123]. This procedure is always indicated after fulfillment of childbearing wishes but it is often offered between 35 and 40 years of age, when many women have not yet dealt with that issue.

Therefore, being a BRCA mutation carrier or being diagnosed with BRCA mutation-related breast cancer presents important difficulties when considering pregnancy and fertility preservation. First of all, these patients seem to present a lower ovarian reserve at baseline compared with non-carriers [124, 125], which would itself represent an obstacle to achieve pregnancy. Moreover, alterations in DNA repair related to BRCA mutations may also make oocytes more vulnerable to chemotherapeutics. These factors combine result in a more marked iatrogenic follicle loss. However, there are some reassuring data concerning safety of pregnancy and fertility treatments for these patients. On one hand, parity and number of children appear to be protective against developing breast cancer BRCA mutation carriers as seen in most large studies [126–129], with some marginal contradictions, apparently due to data analysis [129, 130]. On the other hand, a case–control study did not find an adverse effect of fertility treatment on the risk for developing breast cancer, compared with controls (odds ratio, 1.21; 95% CI, 0.81–1.82).These results must however be regarded with caution, due to the small subgroup sizes [131].

With regard to cancer aggressiveness, BRCA mutation carriers who have already been diagnosed with cancer are more likely to present with triple-negative tumours, which generally have poorer prognosis than other breast cancers. As previously mentioned, because BRCA1 and BRCA2 are involved in DNA repair, it has been suggested that carriers of such mutations may be more sensitive to anticancer agents that act by damaging DNA, such as cisplatin and PARP inhibitors.

Fear of exposure to estrogen limits access to fertility preservation via embryo or oocyte cryopreservation; however, the use of aromatase inhibitors as ovarian stimulants reduces such concern. There does not seem to be a proven ovarian suppression strategy to preserve fertility in women with breast cancer. Ovarian cryopreservation can be used when there is insufficient time to perform ovarian stimulation, since it does not require hormonal stimulation, but this technique is still experimental and it presents safety concerns both in BRCA mutation carriers and in women with hormone receptor-positive tumours. An early oophorectomy can be performed to cryopreserve ovarian tissue from women with BRCA mutations before the risk for ovarian cancer increases with age, but the safety of transplanting this tissue back must be determined [132, 133]. Overall, the last decade has brought many options for women with breast cancer considering fertility preservation, but numerous challenges remain. The presence of BRCA mutations further contributes to these challenges.

Women with BRCA mutations, on the other hand, should be made more aware of fertility issues and also to be motivated to see fertility preservation specialists earlier. These women may also request preimplantation genetic diagnosis (PGD) for BRCA mutations during *in vitro* fertilisation (IVF) to prevent mutation transmission to the embryo [134], although this measure might arise ethical and moral concerns, since BRCA mutations are neither lethal per se nor does their presence guarantee cancer development [83, 135–138].

## Conclusions

Early diagnostic methods, targeted therapies, and prolonged survival rates have made fertility preservation a major issue when treating young breast cancer patients. However, this department is often considered secondary, when early referral and treatment design are crucial to its success. Not only could most young women diagnosed with breast cancer benefit from a wide variety of fertility preservation techniques such as ovarian suppression, oocyte and embryo cryopreservation, immature oocyte retrieval and *in vitro* maturation, and ovarian tissue cryopreservation, but they could resort to these treatments without compromising the efficacy of their anticancer therapy.

Individual characteristics should be taken into account, especially the molecular subtype of breast tumour, which determines the need for gonadotoxic therapy, and the ovarian reserve at the time of diagnosis. Finally, BRCA mutation carriers, either healthy or already diagnosed with breast cancer, are also capable of achieving pregnancy despite their added difficulties -mainly a higher tumour aggressiveness and a lower ovarian reserve.

In summary, both patients and health care professionals involved in breast cancer should keep in mind that pregnancy after breast cancer is possible and that it can be achieved safely for both mother and child.

## Conflicts of interest

The authors declare that they have no conflict of interest.

## Authors’ contributions

María de Pedro performed the literature search. María de Pedro, Borja Otero, and Belén Martín retrieved the information from original articles. Maria de Pedro wrote the sections about breast cancer incidence in premenopausal women, fertility after breast cancer treatment, and special considerations for BRCA-mutation carriers. Borja Otero wrote the sections about evaluation of ovarian reserve and fertility preserving techniques. Belén Martin wrote the sections about fertility-related knowledge, decision making in women with breast cancer, and role of the health care provider in strategies to preserve fertility, and risk of pregnancy and lactation after breast cancer. Maria De Pedro wrote the abstract and revised the first draft of the manuscript. All authors made substantial contributions to the discussion and revised the final version.
